# The promising role of antibody drug conjugate in cancer therapy: Combining targeting ability with cytotoxicity effectively

**DOI:** 10.1002/cam4.4052

**Published:** 2021-06-24

**Authors:** Wen‐Qian Li, Han‐Fei Guo, Ling‐Yu Li, Yong‐Fei Zhang, Jiu‐Wei Cui

**Affiliations:** ^1^ Department of Cancer Center The First Hospital of Jilin University Changchun Jilin China

**Keywords:** antibody drug conjugate, cancer, targeting ability, therapy

## Abstract

**Introduction:**

Traditional cancer therapy has many disadvantages such as low selectivity and high toxicity of chemotherapy, as well as insufficient efficacy of targeted therapy. To enhance the cytotoxic effect and targeting ability, while reducing the toxicity of antitumor drugs, an antibody drug conjugate (ADC) was developed to deliver small molecular cytotoxic payloads directly to tumor cells by binding to specific antibodies via linkers.

**Method:**

By reviewing published literature and the current progress of ADCs, we aimed to summarize the basic characteristics, clinical progress, and challenges of ADCs to provide a reference for clinical practice and further research.

**Results:**

ADC is a conjugate composed of three fundamental components, including monoclonal antibodies, cytotoxic payloads, and stable linkers. The mechanisms of ADC including the classical internalization pathway, antitumor activity of antibodies, bystander effect, and non‐internalizing mechanism. With the development of new drugs and advances in technology, various ADCs have achieved clinical efficacy. To date, nine ADCs have received US Food and Drug Administration (FDA) approval in the field of hematologic tumors and solid tumors, which have become routine clinical treatments.

**Conclusion:**

ADC has changed traditional treatment patterns for cancer patients, which enable the same treatment for pancreatic cancer patients and promote individualized precision treatment. Further exploration of indications could focus on early‐stage cancer patients and combined therapy settings. Besides, the mechanisms of drug resistance, manufacturing techniques, optimized treatment regimens, and appropriate patient selection remain the major topics.

## Graphical abstract

Antibody drug conjugate (ADC) has been under rapid development in recent years, the application of ADC has changed traditional treatment patterns for pan‐cancer patients and has become a great breakthrough for individualized precision treatment. We conducted this review to summarize basic characteristics, clinical progress, and challenges of ADCs to provide a reference for clinical practice and further researches.

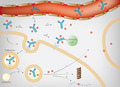

## 1 OVERVIEW

The efficacy of traditional antitumor therapies, which include nonspecific chemotherapy and molecular targeted therapy, is unsatisfactory, owing to the high toxicity of the former and insufficient cytotoxicity and labeling ability of target genes in the latter.^1^ Thus, aiming to combine the strong cytotoxicity of chemotherapy with the high specificity of targeted therapy, antibody drug conjugates (ADCs) are designed to selectively deliver cytotoxic payloads directly to target cancer cells.^2^ Antibody drug conjugates can overcome several traditional problems, including the narrow therapeutic window, low selectivity, and rapid plasma clearance of chemotherapy, as well as the unsatisfactory antitumor efficacy of targeted therapy.^3^ Since 2000, nine ADCs have been approved by the US Food and Drug Administration (FDA) for various treatment settings in both hematologic and solid tumors, and hundreds of studies and clinical trials are currently being explored. However, there are still many challenges in the development of ADC; thus, this review aimed to investigate the current progress and development of ADCs in various types of cancers to provide a reference for clinical applications and further exploration.

## 2 BASIC CHARACTERISTICS OF ADCS

**FIGURE 1:**
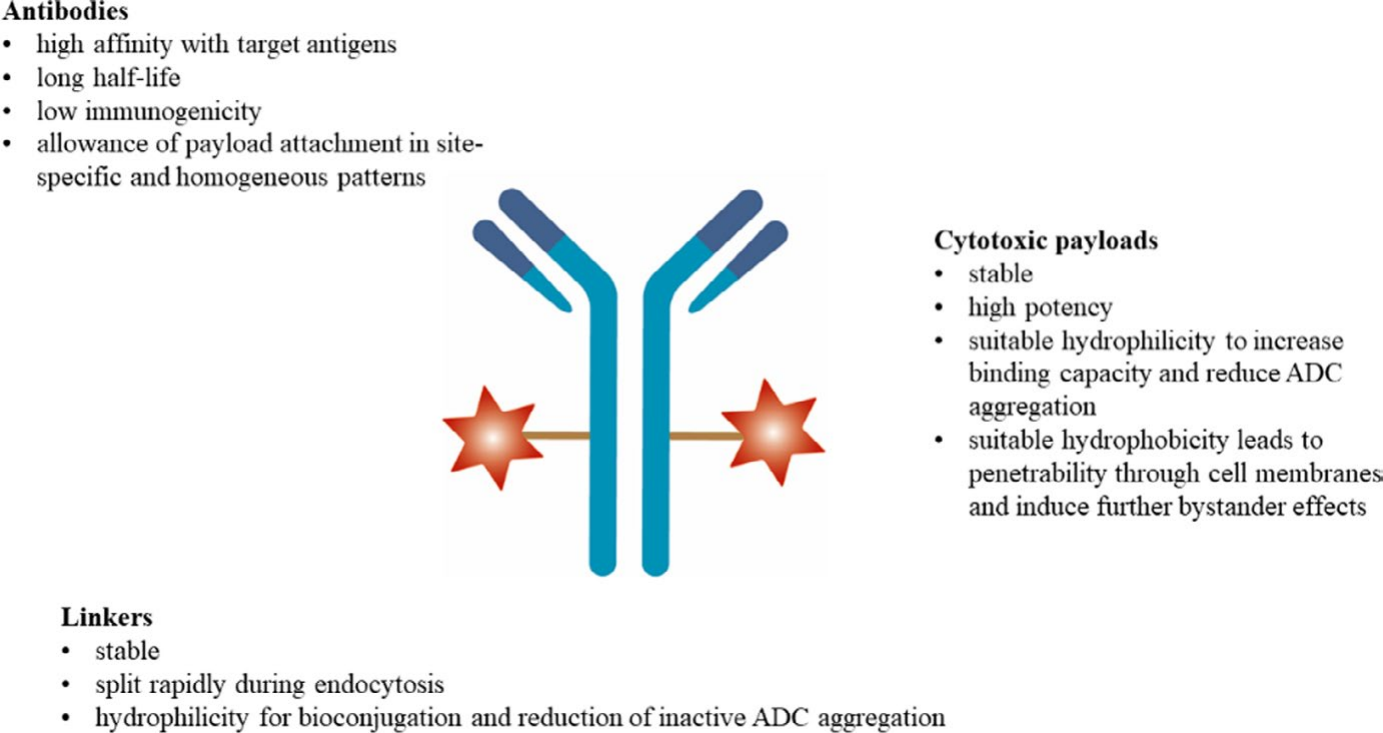
Basic characteristics of ADC. ADC, antibody drug conjugate

Antibody drug conjugate is a conjugate composed of three fundamental components, namely, monoclonal antibodies that target‐specific tumor antigens, high‐potency small molecular cytotoxic payloads, and stable linkers.^4^ The basic characteristics of each component are shown in Figure 1.

Antibody drug conjugate antibodies contain two antigen‐binding fragments (Fabs) that mediate antigen recognition and a constant fragment (Fc) that mediates immune interaction by binding to receptors (FcR) on effector cells.^5^ Appropriate antibodies should have high affinity for target antigens, long half‐life, allowance of site‐specific and homogeneous attachment of payloads, and low immunogenicity to avoid immunoreactions.^6^, ^7^, ^8^ Antibodies are mainly immunoglobulin G (IgG) molecules with high affinity and a long half‐life in the blood circulation system.^9^ The IgG1 isotype is easily produced, with relatively strong antibody‐dependent cytotoxicity (ADCC) and complement‐dependent cytotoxicity (CDC), and is the most commonly used antibody subtype.^10^, ^11^

Cytotoxic payloads are effective components at sub‐nanomolar concentrations with linker‐conjugated functional groups and should be stable in blood. Suitable hydrophilicity is required to increase binding capacity and reduce ADC aggregation, and proper hydrophobicity leads to penetrability through the cell membranes, mediating the bystander effect.^12^, ^13^ The two major categories are microtubule‐targeting agents and DNA‐damaging agents.^14^ Microtubule‐targeting agents can induce cell cycle arrest (G2/M phase) by inhibiting mitotic spindles during chromosome segregation and apoptosis.^15^, ^16^, ^17^ Deoxyribonucleic acid‐damaging agents can induce cell cycle arrest and apoptosis through alkylation, scission, cross‐linking, or intercalation after binding to double helix minor grooves, which have higher cytotoxic efficacies at various cell cycle phases compared with microtubule‐targeting agents.^18^, ^19^, ^20^ Other alternative payloads under development include RNA polymerase inhibitors and spliceosome inhibitors.^13^

Linkers are connections between antibodies and cytotoxic payloads with specific cleavage mechanisms at the target site, which are significant influential factors of pharmacokinetics, pharmacodynamics, and therapeutic windows.^21^, ^22^ Linkers should be stable in plasma to prevent off‐target toxicity due to pyrolysis and should split rapidly during endocytosis to efficiently release payloads.^12^, ^23^ In addition, hydrophilicity is needed for the bioconjugation and reduction of inactive ADC aggregation.^3^, ^8^ Linkers can be categorized as cleavable and non‐cleavable linkers.^24^ Cleavable linkers are the most common type that can exploit the difference in physiological conditions between circulatory and target‐cell conditions.^25^ Non‐cleavable linkers are non‐reducible bonds to amino acid residues on antibodies, and the release of payloads relies on the proteolytic degradation of antibodies in lysosomes; thus, effective internalization and transfer to lysosomes are required.^26^, ^27^ In addition, cleavable linkers can induce bystander effects after the release of cytotoxic payloads, whereas non‐cleavable linkers generally lack a bystander effect because the charged amino acid residues cannot cross cell membranes effectively.^3^, ^12^

## 3 MECHANISMS OF ADCS

The classical mechanism of ADC is as follows: ADCs can specifically bind to target antigens on the cell surface after intravenous injection, and then ADC‐antigen complexes could be internalized via antigen‐dependent endocytosis or antigen‐independent pinocytosis, and endocytosis mediated by clathrin is the major mode.^17^, ^28^ After intracellular trafficking and processing through endosomal and/or lysosomal pathways that rely on organelle acidification,^29^ payloads can be released into the cytoplasm through linker cleavage in the chemical and enzymatic environment or lysosomal proteolytic antibody degradation for non‐cleavable linkers.^30^, ^31^ Payloads play cytotoxic roles by damaging DNA or inhibiting microtubule assembly.^32^, ^33^

**FIGURE 2:**
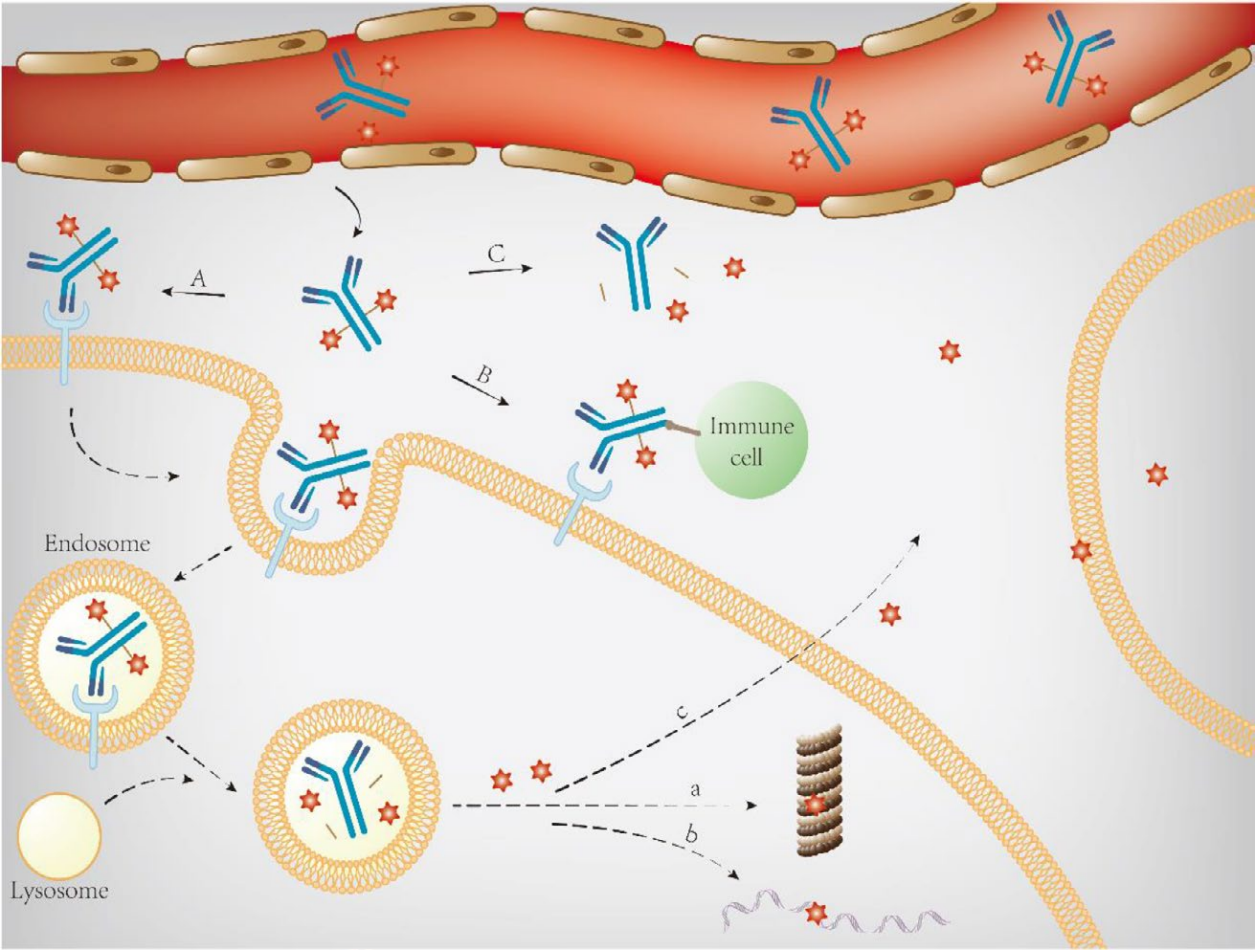
Schematic diagram of ADC mechanism. (A) classical internalizing pathway; (B) retained antitumor activity of antibodies; (C) non‐internalizing pathway; (a) inhibiting microtubule; (b) damaging DNA; (c) bystander effect. ADC, antibody drug conjugate

Antibody drug conjugate can also exert antitumor effects through other mechanisms. First, antibodies could retain the antitumor activity, including the ability to interfere with the function of targets mediated by the Fab region and induce ADCC, CDC, and antibody‐dependent cellular phagocytosis mediated by the Fc region.^34^, ^35^ Second, after ADC molecules are internalized into antigen‐positive tumor cells, cytotoxic payloads with suitable hydrophobicity could permeate cell membranes or be released after the apoptosis of target cells and, subsequently, induce the bystander effect, which can not only kill adjacent tumor cells with negative antigen expression, but also destroy the environment of tumor growth, such as tumor stromal cells and tumor blood vessels.^3^, ^36^ The bystander effect could facilitate the homogenous distribution of payloads and lead to indications for solid tumors with heterogeneously expressed antigens.^37^ Finally, the non‐internalizing mechanism is also under exploration, that is, linker cleavage and payload release could occur extracellularly in the redox and acidic tumor microenvironment with extracellular proteases.^38^, ^39^ A detailed schematic of the mechanism of ADC is shown in Figure 2.

## 4 CLINICAL APPLICATION OF ADCS

Since 2000, five ADCs have received FDA approval for hematologic tumors: gemtuzumab ozogamicin (GO, CD33‐targeting) for acute myeloid leukemia (AML) patients,^40^, ^41^ brentuximab vedotin (BV, CD30‐targeting) for Hodgkin's lymphoma (HL) and non‐Hodgkin's lymphoma (NHL) patients,^42^, ^43^ inotuzumab ozogamicin (INO, CD22‐targeting) for acute lymphoblastic leukemia (ALL) patients,^44^ polatuzumab vedotin‐piiq (PV, CD79b‐targeting) for diffuse large B‐cell lymphoma (DLBCL) patients,^45^ and belantamab mafodotin (BM, B‐cell maturation antigen [BCMA]‐targeting) for multiple myeloma (MM) patients.^46^

In addition, considering that the complex microenvironment of solid tumors hinders the penetration and accessibility of ADCs, the development of ADCs in solid tumors is later than that in hematologic malignancies. Currently, four ADCs have been approved by the FDA in 2013, including three ADCs, ado‐trastuzumab emtansine (T‐DM1, human epidermal growth factor receptor 2 [HER2]‐targeting),^47^ trastuzumab deruxtecan (T‐DXd, HER2‐targeting),^48^, ^49^ and sacituzumab govitecan (SG, trophoblast cell surface antigen 2 [Trop‐2]‐targeting) for breast cancer patients^50^ and enfortumab vedotin (EV, Nectin‐4‐targeting) for urothelial cancer patients.^51^

**FIGURE 3:**
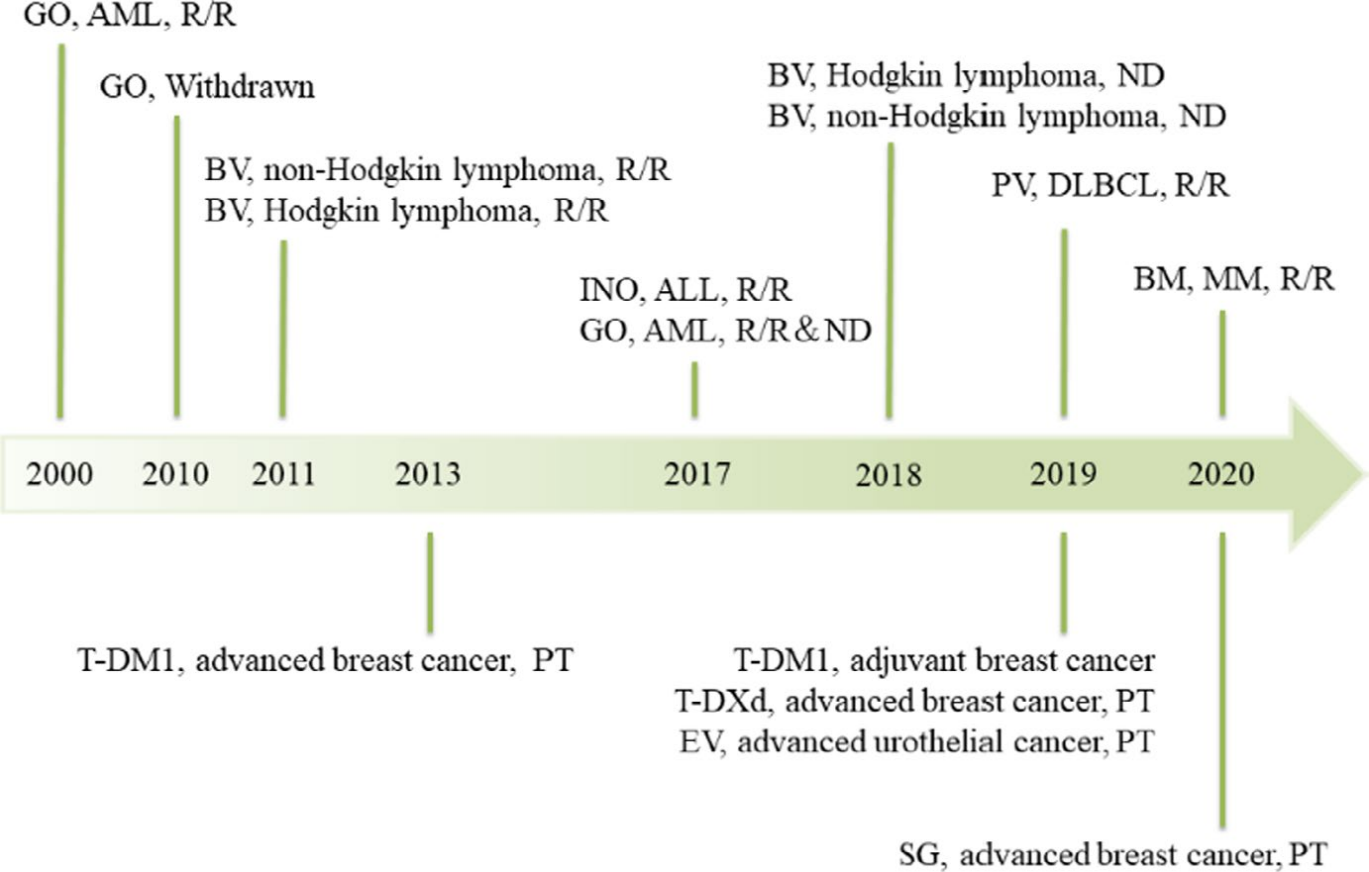
Time sequences and indications of FDA approval. FDA, US Food and Drug Administration

Time sequences and indications for FDA approval are shown in Figure 3. The characteristics and pivotal clinical trials of different ADCs are summarized in Tables 1 and 2, respectively. In the following section, we summarize the application of ADCs in different clinical settings.

**TABLE 1:** Summary of FDA approved ADCs

ADC	Abbreviation/trade name	Time	Antigen	Antibody	Linker	Cytotoxic payload	Mechanism	Approved disease
Gemtuzumab ozogamicin	GO; Mylotarg; CMA‐676	2000/2017	CD33	Humanized IgG4	Cleavable acid‐labile linker	N‐acetyl gamma calicheamicin	DNA‐damaging agents	AML
Brentuximab vedotin	BV; Adcetris; SGN‐35	2011	CD30	Chimeric IgG1	Cleavable protease linker	MMAE	Microtubule‐targeting agents	HL, NHL
Inotuzumab ozogamicin	INO; Besponsa; CMC‐544	2017	CD22	Humanized IgG4	Cleavable acid linker	N‐acetyl gamma calicheamicin	DNA‐damaging agents	ALL
Polatuzumab vedotin‐piiq	PV; Polivy	2019	CD79b	Humanized IgG1	Cleavable protease linker	MMAE	Microtubule‐targeting agents	DLBCL
Belantamab mafodotin	Blenrep; belantamab mafodotin‐blmf; GSK2857916	2020	BCMA	Humanized IgG1	Non‐cleavable protease‐resistant maleimidocaproyl linker	MMAF	Microtubule‐targeting agents	MM
Ado‐trastuzumab emtansine	T‐DM1; Kadcyla	2013	HER2	Humanized IgG1	Non‐cleavable thioether linker	DM1	Microtubule‐targeting agents	Breast cancer
Trastuzumab deruxtecan	T‐DXd; DS‐8201a; [fam‐]trastuzumab deruxtecan‐nxki; Enhertu	2019	HER2	Humanized IgG1	Cleavable tetrapeptide‐based linker	Deruxtecan	DNA‐damaging agents	Breast cancer
Sacituzumab govitecan	SG; Sacituzumab govitecan‐hziy; IMMU‐132; Trodelvy	2020	Trop‐2	Humanized IgG1	Cleavable pH‐sensitive linker	SN‐38	DNA‐damaging agents	Breast cancer
Enfortumab vedotin	EV; enfortumab vedotin‐ejfv; Padcev	2019	Nectin‐4	Fully human IgG1	Cleavable protease linker	MMAE	Microtubule‐targeting agents	Urothelial cancer

Abbreviations: ADC, antibody drug conjugate; AML, acute myeloid leukemia; ALL, acute lymphoblastic leukemia; CD, cluster of differentiation; CTCL, cutaneous T‐cell lymphoma; DLBCL, diffuse large B‐cell lymphoma; DM1, maytansinoids; HL, Hodgkin lymphoma; IgG, immunoglobulin G; MM, multiple myeloma; MMAE, monomethyl auristatin E; MMAF, monomethyl auristatin F; NHL, non‐Hodgkin lymphoma; PTCL, peripheral T‐cell lymphoma; SN‐38, camptothecin analogs.

**TABLE 2:** Pivotal clinical trials of FDA approved ADCs

ADC	Clinical trials	Phase	Line	Regimen	Disease	Drug	ADC dosage	Citation
GO	Sievers, et al	2	First relapse	Monotherapy	AML	GO	9 mg/m^2^, every 14 days	^195^
	SWOG S0106	3	ND	Combined therapy	AML	Induction: DA+GO versus DA; post‐consolidation: GO versus observation	Induction: 6 mg/m^2^, day 4; post‐consolidation: 5 mg/m^2^, every 28 days	^184^
	ALFA‐0701	3	ND	Combined therapy	AML	DA+GO versus DA	Induction: 3 mg/m^2^, days 1, 4, 7; consolidation: 3 mg/m^2^, day 1, 2 cycles	100, 101
	EORTC‐GIMEMA AML‐19	3	ND	Monotherapy	AML	GO versus best supportive care	Induction: 6 mg/m^2^ day 1, 3mg/m^2^ day 8; consolidation: 2 mg/m^2^, monthly	^99^
	MyloFrance‐1	2	First relapse	Monotherapy	AML	GO	3 mg/m^2^, days 1, 4, 7	^54^
	NCT00909168	2	ND	Combined therapy	AML	GO+FLAI	3 mg/m^2^, day 6	^103^
	EORTC‐GIMEM AML‐17	3	ND	Combined therapy	AML	Induction: GO followed by MICE versus MICE; consolidation: GO+ICE versus ICE	Induction: 6 mg/m^2^, days 1 and 15; consolidation: 3 mg/m^2^, day 0	^104^
	SWOG0535	2	ND	Combined therapy	APL	GO+ATRA+ATO	9 mg/m^2^, day 1	^105^
	NCT00143975	2	R/R	Combined therapy	AML	GO+cytarabine+mitoxantrone+ATRA	3 mg/m², day 1	^57^
	NCT00895934	1/2	R/R	Combined therapy	AML	GO+azacytidine+vorinostat	3 mg/m^2^, days 4, 8	^59^
	NCT00766116	1/2	R/R	Combined therapy	AML	GO+azacytidine	6 mg/m^2^, days 7, 21	^60^
	NCT00882102	2	R/R, ND	Combined therapy	AML, MDS	GO+decitabine	3 mg/m^2^, day 5	^58^
BV	NCT00848926	2	R/R	Monotherapy	HL	BV	1.8 mg/kg, every 21 days, 16 cycles	65, 69
	AETHERA	3	Consolidation therapy after ASCT	Monotherapy	HL	BV	1.8 mg/kg, every 21 days	67, 68
	NCT01393717	2	R/R	Monotherapy	HL	BV	1.8 mg/kg, every 21 days	^70^
	NCT02243436	1/2	R/R	Combined therapy	HL	BV+ESHAP	1.8 mg/kg, day 1, every 21 days	^71^
	NCT02227199	1/2	R/R	Combined therapy	HL	BV+ICE	1.5 mg/kg, days 1, 8, every 21 days	^72^
	NCT01874054	1/2	R/R	Combined therapy	HL	BV+bendamustine	1.8 mg/kg, day 1, every 21 days	^73^
	NCT02280993	2	R/R	Combined therapy	HL	BV+DHAP	1.8 mg/kg, day 1, every 21 days	^74^
	NCT02572167	1/2	R/R	Combined therapy	HL	BV+nivolumab	1.8 mg/kg, day 1, every 21 days, 4 cycles	^75^
	ECHELON‐1	3	ND	Combined therapy	HL	BV+AVD versus ABVD	1.2 mg/kg, days 1, 15, every 28 days, 6 cycles	^106^
	NCT00866047	2	R/R	Monotherapy	sALCL	BV	1.8 mg/kg, every 21 days, 16 cycles	76, 77
	NCT01421667	2	R/R	Monotherapy	NHL	BV	1.8 mg/kg, every 21 days	^78^
	ALCANZA	3	R/R	Monotherapy	CTCL	BV	1.8 mg/kg, every 21 days, 16 cycles	^79^
	ECHELON‐2	3	ND	Combined therapy	PTCL	BV+CHP versus CHOP	1.8 mg/kg, every 21 days, 6–8 cycles	^108^
	CheckMate 436	1/2	R/R	Combined therapy	PMBL	BV+nivolumab	1.8 mg/kg, every 21 days	^81^
	NCT01925612	2	ND	Combined therapy	DLBCL	BV+R‐CHP	1.8 mg/kg, every 21 days, 6 cycles	^111^
	NCT01994850	1/2	ND	Combined therapy	B‐cell lymphoma	BV+R‐CHP	1.8 mg/kg, every 21 days, 6 cycles	^112^
INO	INOVATE	3	R/R	Monotherapy	ALL	INO	Total 1.8 mg/m² per cycle: 0.8 mg/m² on day 1; 0.5 mg/m² on day 8, day 15. Cycle 1, 21 days; subsequent cycles, 28 days. For patients achieving CR: 0.5 mg/m², days 1, 8, and 15	83, 84, 85
	NCT01371630	2	ND /salvage	Combined therapy	ALL	INO+mini‐hyper‐CVD	1.3–1.8 mg/m² in cycle 1, 1.0–1.3 mg/m² in cycle 2–4, every 4 weeks	86, 87, 113
	NCT00299494	1/2	R/R	Combined therapy	NHL	INO+rituximab	1.8 mg/m², every 28 days, 8 cycles	^88^
	NCT01232556	3	R/R	Combined therapy	NHL	INO+rituximab versus chemotherapy (bendamustine or gemcitabine)+rituximab	1.8 mg/m², every 28 days, 3–6 cycles	^91^
PV	GO29365	1/2	R/R	Combined therapy	DLBCL	PV+bendamustine+rituximab	1.8 mg/kg, every 21 days	^93^
	NCT01992653	1/2	ND	Combined therapy	DLBCL	PV+rituximab or obinutuzumab+CHP	1.8 mg/kg, every 21 days	^115^
BM	DREAMM‐2	2	PT	Monotherapy	MM	BM	2.5 or 3.4 mg/kg, every 3 weeks	^95^
T‐DM1	EMILIA	3	PT	Monotherapy	Breast cancer	T‐DM1	3.6 mg/kg, every 21 days	121, 122, 123
	TH3RESA	3	PT	Monotherapy	Breast cancer	T‐DM1	3.6 mg/kg, every 21 days	126, 127
	NCT02236000	1b	PT	Combined therapy	Breast cancer	T‐DM1+Neratinib	3.6 mg/kg, every 21 days	^128^
	MARIANNE	3	ND	Monotherapy / Combined therapy	Breast cancer	T‐DM1/T‐DM1+pertuzumab	3.6 mg/kg, every 21 days	^146^
	KRISTINE	3	Neoadjuvant	Combined therapy	Breast cancer	T‐DM1 plus pertuzumab	3.6 mg/kg, every 21 days	151, 152
	KATHERINE	3	Adjuvant	Monotherapy	Breast cancer	T‐DM1	3.6 mg/kg, every 21 days	^149^
	KAMILLA trial	3	PT	Monotherapy	Breast cancer	T‐DM1	3.6 mg/kg, every 21 days	^125^
T‐DXd	DESTINY‐Breast01	2	PT	Monotherapy	Breast cancer	T‐DXd	5.4 mg/kg, every 21 days	^134^
	DESTINY‐Gastric01/NCT03329690	2	PT	Monotherapy	Gastric cancer	T‐DXd	6.4 mg/kg, every 21 days	^135^
	NCT02564900	1	PT	Monotherapy	Breast cancer	T‐DXd	6.4 mg/kg, every 21 days	^136^
SG	NCT01631552	1/2	PT	Monotherapy	Breast cancer	IMMU‐132	8 or 10 mg/kg, days 1 and 8, every 21 days	138, 139, 140, 141, 142, 143
EV	EV‐101	1	PT	Monotherapy	Urothelial cancer	EV	1.25 mg/kg, days 1, 8, 15, every 29 days	^144^
	EV‐201	2	PT	Monotherapy	Urothelial cancer	EV	1.25 mg/kg, days 1, 8, 15, every 28 days	^145^

Abbreviations: ABVDH, doxorubicin+bleomycin+vinblastine+dacarbazine; ADC, antibody drug conjugate; ALL, acute lymphoblastic leukemia; AML, acute myeloid leukemia; APL, acute promyelocytic leukemia; ATO, arsenic trioxide; ATRA, all‐trans retinoic acid; AVD, doxorubicin+vinblastine+dacarbazine; BM, belantamab mafodotin; BV, brentuximab vedotin; CHOP, cyclophosphamide+doxorubicin+vincristine+prednisone; CHP, cyclophosphamide+doxorubicin+prednisone; CR, complete remission; CTCL, cutaneous T‐cell lymphoma; DA, daunorubicin+cytarabine; DHAP, dexamethasone+cisplatin+cytarabine; DLBCL, diffuse large B‐cell lymphoma; ESHAP, etoposide+solumedrol+high‐dose AraC+cisplatin; EV, enfortumab vedotin; FLAI, fludarabine+cytarabine+idarubicin; GO, gemtuzumab ozogamicin; HL, Hodgkin lymphoma; ICE, ifosfamide+carboplatin+etoposide; INO, inotuzumab ozogamicin; MDS, myelodysplastic syndrome; MICE, mitoxantrone+etoposide+cytarabine; mini‐hyper‐CVD, cyclophosphamide+vincristine+methotrexate+cytarabine; MM, multiple myeloma; ND, newly diagnosed; NHL, non‐Hodgkin lymphoma; PMBL, primary mediastinal B‐cell lymphoma; PT, previously treated; PTCL, peripheral T‐cell lymphoma; PV, polatuzumab vedotin‐piiq; R‐CHP, rituximab+cyclophosphamide+doxorubicin+prednisone; R/R, relapsed/refractory; sALCL, systemic anaplastic large‐cell lymphoma; SG, sacituzumab govitecan; T‐DM1, ado‐trastuzumab emtansine; T‐DXd, trastuzumab deruxtecan.

### 4.1 Hematologic malignancies

#### 4.1.1 Relapsed or refractory cancer

First, anti‐CD33 GO was explored mostly in AML patients. Acute myeloid leukemia is a common type of aggressive hematologic malignancy characterized by abnormal proliferation and differentiation of immature myeloid cells, which accounts for 20% of hematologic malignancy‐related deaths.^52^, ^53^ The fractionated dosage of GO was observed in the phase 2 MyloFrance‐1 trial for the first Relapsed or refractory (R/R) CD33‐positive AML patients; 26% of patients achieved complete remission (CR) with a median relapse‐free survival (RFS) of 11 months and manageable toxicities, which led to FDA approval.^54^ Several studies explored the efficacy of GO in combination with chemotherapy as salvage therapy, including the combination of GO and DA therapy (daunorubicin plus cytarabine) (overall response rate [ORR]: 38.8%; CR rate: 22.2%; 2‐year RFS rate: 18.5%; 2‐year overall survival [OS] rate: 26%),^55^ the combination of GO and MYLODAM schema (cytarabine and mitoxantrone) (ORR: 67%; 2‐year RFS rate: 36%; 2‐year OS rate: 54%),^56^ the combination of GO and high‐dose cytarabine, mitoxantrone, and all‐trans retinoic acid (ATRA) (CR/CR with incomplete hematologic recovery [CRi] rate: 51%; ORR: 61.5%; 4‐year OS rate: 32%),^57^ as well as the combination of GO and decitabine (CR/CRi rate: 18%; median OS: 3.5 months).^58^ Moreover, two phase 1/2 trials showed the enhanced efficacy of hypomethylating agent therapy in addition to GO through epigenetic effects in R/R AML patients (NCT00766116 trial, GO, plus azacytidine; CR/CRi rate: 24%; NCT00895934 trial, GO, azacytidine, plus vorinostat; CR/CRi rate: 41.9%; median OS: 224.5 days).^59^, ^60^ Furthermore, a retrospective study also showed the efficacy of GO combined with intermediate‐dose cytarabine for relapsed patients after stem‐cell transplantation (ORR: 60%; median OS: 103 days; median EFS: 76 days).^61^ Consequently, GO‐based regimens might be considered as a salvage and bridge therapy to transplant for R/R AML patients and may also be a potential therapy for patients after transplantation.

Second, anti‐CD30 BV was detected in both HL and NHL patients. Hodgkin's lymphoma is a relatively rare B‐cell malignancy that contributes to 10% of lymphomas, while NHL is common and contains a series of heterogeneous malignancies with various pathological and clinical characteristics.^62^, ^63^, ^64^ The phase 2 NCT00848926 trial showed the efficacy of monotherapy BV for R/R CD30‐positive HL patients after an autologous stem‐cell transplantation (ASCT) or at least two lines of multiagent chemotherapies (ORR: 75%; CR rate: 34%; adverse event [AE] over grade 3: 55%; 5‐year progression‐free survival [PFS] rate: 22%; 5‐year OS rate: 41%).^65^, ^66^ The phase 3 AETHERA trial also demonstrated the efficacy of BV compared with placebo as consolidation therapy for HL patients at high risk of R/R after ASCT (median PFS: 42.9% vs. 24.1%; *p* = 0.0013; 5‐year PFS rate: 59% vs. 41%; hazard ratio [HR]: 0.521; 95% confidence interval [CI]: 0.379–0.717).^67^, ^68^ Based on these two trials, BV received FDA approval for patients with R/R CD30‐positive HL and patients with a high risk of R/R after ASCT.^42^, ^69^ For R/R CD30‐positive HL patients prior to ASCT, the phase 2 NCT01393717 study demonstrated that an ORR of 68%, besides, 49% of patients received ASCT without salvage chemotherapy.^70^ In addition, in terms of combination therapy, the combination of BV and chemotherapy was explored widely for R/R HL patients before ASCT, with an approximate ORR of 90% and a CR rate in the range of 70–80%, including BV plus etoposide, solumedrol, high‐dose AraC, and cisplatin (ESHAP, NCT02243436 trial)^71^; BV plus ifosfamide, carboplatin, and etoposide (ICE, NCT02227199 trial)^72^; BV plus bendamustine (NCT01874054 trial)^73^; and BV plus dexamethasone, cisplatin, and cytarabine (DHAP, NCT02280993 trial).^74^ Moreover, a phase 1/2 NCT02572167 trial investigated the combination of BV and immunotherapy as initial salvage therapy for patients with R/R HL; 82% of patients receiving BV plus nivolumab achieved ORR and 61% achieved CR. Although 98% of patients had AEs, the majority were in grades 1–2.^75^ Thus, the combination therapy of BV showed activity with manageable AEs for R/R HL patients prior to ASCT and should be further confirmed in phase 3 trials.

The phase 2 NCT00866047 trial showed the efficacy of BV monotherapy for previously treated CD30‐positive systemic anaplastic large cell lymphoma (sALCL) patients (CR rate: 66%; median PFS: 20.0 months; 5‐year PFS: 39%; 5‐year OS rate: 60%).^76^, ^77^ In the phase 2 NCT01421667 study, patients with R/R CD30‐positive peripheral T‐cell lymphoma (PTCL) receiving BV achieved an ORR of 41%; for patients with angioimmunoblastic T‐cell lymphoma, the ORR was 54%, and the median PFS was 6.7 months.^78^ In addition, the phase 3 ALCANZA trial explored the benefit of BV on R/R CD30‐positive cutaneous T‐cell lymphomas patients; patients receiving BV had a superior clinical benefit compared with the control group (methotrexate or bexarotene), including ORR (67% vs. 20%; *p* < 0.0001), complete response rate (16% vs. 2%; *p* = 0.0046), and median PFS (17.2% vs. 3.5%; *p* < 0·0001). The most frequently reported AE is peripheral sensory neuropathy.^79^ Subsequently, BV therapy has been approved by the FDA for NHL.^42^, ^43^ For B‐cell NHL, the subset of phase 2 NCT01421667 study showed the efficacy of BV for patients with R/R CD30‐positive B‐cell NHL, mainly DLBCL, with an ORR of 44% and a CR rate of 17%.^80^ The combination therapy was detected in the phase 1/2 CheckMate436 trial; the combination of BV and nivolumab for R/R CD30‐positive primary mediastinal B‐cell lymphoma (PMBL) patients with an ORR of 73%, a CR rate of 37%, besides, 53% of patients developed grade 3–4 AEs ^81^ AEs.

Third, CD22‐directed INO was applied in patient with ALL, a heterogeneous neoplasm of lymphoid progenitors, which consists of 85% B‐cell lineage and 15% T‐cell lineage. Patients with ALL have a poor prognosis, especially in adults.^82^ Inotuzumab ozogamicin was explored as a monotherapy for adult R/R CD22‐positive B‐cell precursor ALL patients in the phase 3 INOVATE trial. Results demonstrated that INO group achieved a better CR rate (80.7% vs. 29.4%; *p* < 0.001), median PFS (5.0 vs. 1.8 months; *p* < 0.001), and median OS (7.7 vs. 6.7 months; *p* = 0.04) compared with the chemotherapy group.^83^ A long‐term survival report showed sustained benefit (CR/CRi rate: 73.8% vs. 30.9%, *p* < 0.0001; median OS: 7.7 vs. 6.2 months, *p* = 0.0105), despite a higher incidence of hepatotoxicities (51% vs. 34%).^84^, ^85^ Thus, INO received FDA approval for adult patients with R/R B‐cell precursor ALL.^44^ In addition, the application of INO in combination therapy was explored in both patients with ALL and B‐cell NHL. The phase 2 NCT01371630 trial found the efficacy of INO plus mini‐hyper‐CVD chemotherapy regimen (cyclophosphamide, vincristine, methotrexate, an cytarabine) for Philadelphia chromosome‐negative R/R ALL patients (ORR: 78%; CR rate: 59%; median RFS: 8 months; median OS: 11 months),^86^ while for patients in the first relapse, blinatumomab was also an additional option for combination therapy (ORR: 92%; CR rate: 73%; median RFS: 11 months; median OS: 25 months).^87^ For B‐cell NHL, the phase 1/2 NCT00299494 trial investigated the efficacy of INO plus rituximab for R/R CD20/CD22‐positive B‐cell NHL patients; the ORR was 87% and 74%, and the 2‐year PFS rates were 68% and 42% for follicular lymphoma (FL) and DLBCL, respectively.^88^ Efficacy was also found in the combination therapy of INO with both rituximab, gemcitabine, dexamethasone, and cisplatin (R‐GDP) as well as rituximab, cyclophosphamide, vincristine, and prednisone (R‐CVP) in R/R CD22‐positive B‐cell NHL patients in a phase 1 trial (NCT01055496).^89^, ^90^ However, phase 3 NCT01232556 trial compared INO plus rituximab with chemotherapy plus rituximab for R/R aggressive B‐cell NHL, and no significant benefit was shown, while two patients suffered from grade 3 veno‐occlusive disease (VOD)/sinusoidal obstruction syndrome (SOS).^91^

Fourth, CD79b‐targeted PV was explored in patients with DLBCL. Diffuse large B‐cell lymphoma contributes to approximately 25% of NHL cases, and despite its curability, 40% of patients suffer from R/R disease.^92^ The combination therapy of PV plus bendamustine and rituximab (pola‐BR) was evaluated in comparison with bendamustine and rituximab (BR) in a randomized phase 1b/2 GO29365 trial for patients with transplantation‐ineligible R/R DLBCL. Results showed that the pola‐BR group achieved superior CR rate (40.0% vs. 17.5%, *p* = 0.026), PFS (median PFS: 9.5 vs. 3.7 months, *p* < 0.001), and OS (median OS: 12.4 vs. 4.7 months; *p* = 0.002), while higher incidences were found in grades 3–4 neutropenia, anemia, and thrombocytopenia.^93^ The results led to the accelerated FDA approval of PV combined with bendamustine plus rituximab for R/R adult patients with DLBCL after two previous treatments.^45^

Finally, BCMA‐targeted BM was investigated in bone marrow cancer and MM patients. Although the 5‐year survival rate of MM patients is nearly 70%, more than 10% of patients still have a poor prognosis.^94^ The phase 2 DREAMM‐2 trial showed benefits for R/R MM patients after at least four previous therapies, including a proteasome inhibitor, an anti‐CD38 monoclonal antibody, and an immunomodulatory drug (BM at 2.5 mg/kg every 3 weeks; ORR: 31%; median PFS: 2.9 months; BM at 3.4 mg/kg every 3 weeks; ORR: 34%; median PFS: 4.9 months) with a most common AE of keratopathy (27%; 21%).^95^ Thus, BM has received FDA approval for R/R MM patients.^46^ In addition, efficacies were also found in combined therapies, the phase 1/2 DREAMM‐4 trial (BM+pembrolizumab) showed an ORR of 67% (2.5 mg/kg cohort) and 43% (3.4 mg/kg cohort) for R/R MM patients receiving ≥3 previous therapy; the phase 1/2 DREAMM‐6 study (BM [2.5 mg/kg]+bortezomib/dexamethasone) showed an ORR of 78% for R/R MM patients receiving ≥1 previous therapy.^96^, ^97^ Several phase 3 trials are ongoing to verify the benefits of both monotherapy and combined therapies (DREAMM‐3, DREAMM‐7, DREAMM‐8, and DREAMM‐9).^98^

#### 4.1.2 Newly diagnosed cancer

First, the phase 3 EORTC‐GIMEMA AML‐19 trial explored the front‐line monotherapy of GO compared with best supportive care for older AML patients who were ineligible for intensive chemotherapy. The results showed that the administration of GO achieved a CR/CRi rate of 27% and a superior OS benefit (median OS: 4.9 vs. 3.6 months, *p* = 0.005) compared with that of the control group with similar serious AE rates.^99^ The combination therapy of GO with chemotherapy for older patients with newly diagnosed AML was explored in the phase 3 ALFA‐0701 trial. Comparing with the standard DA induction therapy, the combination of GO with DA showed a significant event‐free survival (EFS) benefit (median EFS: 17.3 vs. 9.5 months, *p* = 0.0002), but there was no significant OS benefit. Manageable AEs were observed with a fractionated dosage of GO.^100^, ^101^ A meta‐analysis further confirmed the efficacy of GO plus standard induction chemotherapy for AML patients, which showed a significantly superior OS (5‐year OS rate: 34.6% vs. 30.7%, *p* = 0.01), especially for patients with favorable cytogenetics or intermediate risk.^102^ Thus, the FDA approved both monotherapy and combined therapy of GO for adult patients with CD33‐positive untreated AML in September 2017.^40^, ^41^

In addition, other combination regimens included the combination of GO and FLAI regimen (fludarabine, cytarabine, and idarubicin) (CR rate: 82%; 5‐year OS rate: 52%),^103^ the combination of GO and decitabine (CR/CRi rate: 45%; median OS: 7 months),^58^ and the combination of GO and MICE regimen (mitoxantrone, etoposide, and cytarabine). No survival benefits were observed, but higher mortality rates compared with that of chemotherapy were recorded.^104^ Furthermore, a phase 2 study (SWOG0535 trial) showed the benefits and tolerances of GO, ATRA, and arsenic trioxide in newly diagnosed high‐risk acute promyelocytic leukemia patients (CR rate: 86%; 3‐year EFS rate: 78%; 3‐year OS rate: 86%; 6‐week mortality rate: 11%).^105^ Thus, GO with various regimens may be an option for newly diagnosed AML patients.

Second, the first‐line setting of BV was explored in both HL and NHL. The randomized phase 3 ECHELON‐1 trial explored the efficacy of the combination of BV and chemotherapy for previously untreated stage III/IV classic HL patients and led to an FDA approval. Patients receiving BV, doxorubicin, vinblastine, and dacarbazine (A+AVD) were compared with those receiving doxorubicin, bleomycin, vinblastine, and dacarbazine (ABVD); the results showed a significant benefit for PFS (2‐year PFS rate: 82.1% vs. 77.2%; *p* = 0.04), despite a higher rate of AEs over grade 3 (83% vs. 66%).^106^, ^107^ For NHL patients, the phase 3 ECHELON‐2 trial showed that the combination group (BV, cyclophosphamide, doxorubicin, and prednisone [A+CHP]) also achieved clinical benefit in terms of PFS (median PFS: 48.2 vs. 20.8 months; *p* = 0.0110) and OS (HR: 0.66; 95% CI: 0.46–0.95; *p* = 0.0244) compared with chemotherapy (cyclophosphamide, doxorubicin, vincristine, and prednisone) for patients with treatment‐naive CD30‐positive PTCL,^108^ which led to an FDA approval.^109^, ^110^ In addition, the phase 2 NCT01925612 trial explored the first‐line application of BV in combination with rituximab, cyclophosphamide, doxorubicin, and prednisone (R‐CHP) for high‐intermediate/high‐risk DLBCL patients, and the results showed an ORR of 91%, while for CD30^+^ patients, the 18‐month PFS rate was 79% and the OS rate was 92%.^111^ Another phase 1/2 NCT01994850 trial showed an ORR of 100% when combining BV with R‐CHP as first‐line therapy for CD30‐positive B‐cell lymphoma patients, with a CR rate of 86% and a 2‐year PFS and OS of 85% and 100%, respectively.^112^

Third, INO was explored in the first‐line setting in the phase 2 NCT01371630 trial for Philadelphia chromosome‐negative ALL patients aged over 60 years, and the application of INO plus mini‐hyper‐CVD chemotherapy regimen showed a 2‐year PFS rate of 59%, with manageable toxicities. The most common grade 3–4 AEs were prolonged thrombocytopenia, and VOD occurred in 8% of patients.^113^ Veno‐occlusive disease should be considered for patients with abnormal liver function during the administration of INO, while the application of blinatumomab in consolidation therapy could prolong the duration between INO and ASCT, which might decrease the VOD risk.^114^

Fourth, in terms of PV, the phase 1b–2 trial (NCT01992653) investigated the combination therapy of PV in addition to rituximab or obinutuzumab plus CHP in treatment‐naïve DLBCL patients, with a complete response rate of 77% and an ORR of 89%.^115^ The treatment regimen was validated in the Phase 3 POLARIX trial.

### 4.2 Solid tumors

The development of ADC in solid tumors has mainly focused on breast and urothelial cancers.

Breast cancer is the second most common cancer, with various subtypes according to histopathology and the expression of both hormone receptors and growth factors.^116^, ^117^ Urothelial carcinoma is the major type of bladder cancer, and it can occur in the upper urinary tract and proximal urethra.^118^, ^119^

#### 4.2.1 Previously treated advanced cancer

The HER2‐targeted ADC, T‐DM1, was the first FDA‐approved ADC in solid tumors. The FDA approval of T‐DM1 for previously treated (trastuzumab and a taxane) patients with HER2‐positive metastatic breast cancer was based on the phase 3 EMILIA trial.^47^, ^120^ Results showed patients receiving T‐DM1 had a significant prolonged PFS (median PFS: 9.6 months vs. 6.4 months; *p* < 0.001) and OS (median OS: 30.9 months vs. 25.1 months; *p* < 0.001) compared with lapatinib plus capecitabine group, as well as less AEs of grade 3 or above (41% vs. 57%).^121^ The final descriptive analysis showed sustained benefit (29.9 months vs. 25.9 months; HR: 0.75; 95% CI: 0.64–0.88).^122^ In addition, subgroup analysis showed that T‐DM1 could penetrate the blood–brain barrier and a significantly superior OS benefit was observed for patients with brain metastases (median OS: 26.8 months vs. 12.9 months, *p* = 0.008) for brain metastases patients.^123^, ^124^ The efficacy for brain metastases was further confirmed in the phase 3 KAMILLA trial (ORR: 21.4%; clinical benefit rate [CBR]: 42.9%; median PFS: 5.5 months; median OS: 18.9 months).^125^ Additionally, in the phase 3 TH3RESA study, T‐DM1 was compared with the treatment of the physician's choice in advanced HER2‐positive breast cancer patients who had previously received at least two HER2‐directed agents. Significant superior survival was shown in patients receiving T‐DM1 (median PFS: 6.2 months vs. 3.3 months, *p* < 0.0001; median OS: 22.7 months vs. 15.8 months, *p* = 0.0007), with a lower rate of AEs over grade 3 (40% vs. 47%) but a higher rate of serious AEs (25% vs. 22%).^126^, ^127^ The survival benefits and acceptable toxicity suggested that T‐DM1 could be used as a posterior line therapy for advanced HER2‐positive breast cancer. Moreover, new combination therapies are still being explored, including T‐DM1 combined with neratinib, which showed efficacy (ORR: 63%) in a phase 1b trial (NCT02236000) and needed further investigation.^128^

In addition, T‐DM1 was also investigated in other HER2‐positive solid tumors, including gastric cancer (GATSBY trial, T‐DM1 vs. taxane, without superior benefit) ^129^, ^130^ and lung cancer (NCT02675829 trial, PR rate: 44%, median PFS: 5 months; NCT02289833 trial, ORR: 20%; CBR: 30%).^131^, ^132^ Further studies have focused on other solid cancers, including bladder cancers, urinary tract cancers, pancreatic cancer, and colorectal cancers, and combined therapies of T‐DM1 and immunotherapy have also been explored.

Second, for HER2‐targeted T‐DXd, the payload of T‐DXd (deruxtecan) could be released and permeate cell membranes, inducing effective cytotoxicity on neighboring tumor cells despite the expression levels of HER2, which expanded its indications for tumors with heterogeneous HER2 expression.^133^ In phase 2, the DESTINY‐Breast01 trial explored the efficacy of T‐DXd for advanced HER2‐positive breast cancer patients who were heavily pre‐treated and already received T‐DM1; the median PFS was 16.4 months, with 60.9% of patients responding; interstitial lung disease needed extra attention and was observed in 13.6% of the patients.^134^ Based on these results, T‐DXd received accelerated FDA approval for advanced HER2‐positive breast cancer patients who received at least two lines of anti‐HER2‐based regimens in December 2019.^48^, ^49^

The efficacy of T‐DXd has also been observed in other types of tumors. The phase 2 DESTINY‐Gastric01 trial showed the benefit of T‐DXd compared with chemotherapy for previously treated patients with HER2‐positive advanced gastric cancer (ORR: 51% vs. 14%, *p* < 0.001; median OS: 12.5 months vs. 8.4 months, *p* = 0.01).^135^ In addition, T‐DXd was also evaluated in pretreated, HER2‐expressing, or HER2‐mutant advanced solid tumors (including non‐small cell lung cancer [NSCLC], colorectal cancer, and other solid cancers) in the phase 1 NCT02564900 trial. For the entire population, an ORR of 28.3% and a median PFS of 7.2 months were found, while HER2‐mutant NSCLC patients showed an ORR of 72.7% and a median PFS of 11.3 months.^136^ In addition, several phase 3 trials are ongoing, including the confirmation of T‐DXd for breast cancer patients as well as the exploration of T‐DXd for patients with HER‐low expression and combination therapies.^48^

Third, for the Trop‐2‐targeted SG, the novel pH‐sensitive hydrolyzable CL2A linker enabled SG to release SN‐38 in both intracellular tumors and the tumor microenvironment, which could induce bystander effects.^137^ The phase 1/2 NCT01631552 trial in small cell lung cancer (SCLC) patients with advanced epithelial cancers ^138^ and metastatic urothelial carcinoma patients (ORR: 33.3%; CBR: 45.4%; median PFS: 5.5 months; median OS: 13.0 months) ^139^ were hormone receptor‐positive, while metastatic urothelial carcinoma patients (ORR: 31%; CBR: 44.4%; median PFS: 5.5 months; median OS: 12 months)^140^; metastatic urothelial carcinoma patients (ORR: 31%; CBR: 47%; median PFS: 7.3 months; median OS: 18.9 months)^141^; metastatic NSCLC patients (ORR: 19%; CBR: 43%; median PFS: 5.2 months; median OS: 9.5 months)^142^; and metastatic SCLC patients (ORR: 14%; CBR: 34%; median PFS: 3.7 months; median OS: 7.5 months) were HER2‐negative.^143^ Thus, with durable efficacy and tolerable AEs, SG received accelerated FDA approval for metastatic triple‐negative breast cancer patients after at least two lines of therapies in 2020, and fast‐track designations were also granted for patients with metastatic urothelial carcinoma, NSCLC, and SCLC.^50^ Further confirmatory and exploratory trials are ongoing, including the phase 3 ASCENT trial for breast cancer patients and phase 2 studies for urothelial cancer as well as other endometrial cancer patients. In the meantime, combination therapies of SG are also being explored.

Furthermore, the Nectin‐4‐directed EV was first evaluated in the phase 1 study EV‐101 trial for heavily pretreated advanced urothelial carcinoma patients, with an ORR of 43% and a median OS of 12.3 months with tolerable AEs.^144^ The FDA approval was based on the results of the phase 2 EV‐201 trial for advanced urothelial cancer patients after treatment with platinum‐containing chemotherapy and anti‐PD‐1/L1 therapy. Efficacy was found with an ORR of 44%, a median PFS of 5.8 months, and a median OS of 11.7 months, wherein the most common AEs were fatigue and peripheral neuropathy.^51^, ^145^ A confirmation phase 3 EV‐301 trial exploring EVs compared with chemotherapy is ongoing.

#### 4.2.2 Newly diagnosed advanced cancer

First‐line application of TDM‐1 was evaluated for advanced HER2‐positive breast cancer patients, wherein the phase 3 MARIANNE trial compared T‐DM1 plus pertuzumab and T‐DM1 monotherapy with trastuzumab plus taxane (control group). Results showed that T‐DM1 with or without pertuzumab had similar efficacy with the control group (median PFS: 15.2 months vs. 14.1 months vs. 13.7 months; response rate: 64.2% vs. 59.7% vs. 67.9%; median response duration: 21.2 months vs. 20.7 months vs. 12.5 months), while there was a lower rate of AEs over grade 3 (46.2% vs. 45.4% vs. 54.1%).^146^ Considering the longer duration of response and safety of T‐DM1, the National Comprehensive Cancer Network recommended first‐line T‐DM1 for HER2‐positive breast cancer patients who were not candidates for preferred standard treatment.^147^

Besides, first‐line applications of other ADCs were under investigation, the ongoing phase 1 EV‐103 trial explored the combination therapy of EV and chemotherapy or immunotherapy as first‐line therapy for advanced urothelial cancer patients, with a 71% ORR for patients receiving EV and pembrolizumab.^148^

#### 4.2.3 Early stage cancer

For early stage cancer, TDM‐1 was explored in both adjuvant and neoadjuvant settings. In phase 3, the KATHERINE trial explored the application of adjuvant T‐DM1 compared with trastuzumab for HER2‐positive early breast cancer patients with residual invasive disease after the administration of neoadjuvant chemotherapy and HER2‐targeted therapy. Patients receiving T‐DM1 achieved better invasive disease‐free survival (iDFS) than patients receiving trastuzumab (3‐year iDFS rate: 88.3% vs. 77.0%). The application of adjuvant T‐DM1 reduced the risk of recurrence or death by 50% (*p* < 0.001), but a higher rate of AEs of grade 3 (25.7% vs. 15.4%).^149^ Thus, the FDA approved T‐DM1 as adjuvant treatment for HER2‐positive early stage breast cancer patients with residual invasive disease, which further expanded the indications for T‐DM1.^150^ In terms of neoadjuvant therapy for HER2‐positive operable breast cancer patients, the phase 3 KRISTINE trial explored T‐DM1 plus pertuzumab (T‐DM1+P, also as adjuvant therapy) versus docetaxel, carboplatin, and trastuzumab plus pertuzumab (TCH+P, followed by adjuvant trastuzumab plus pertuzumab), and patients in the T‐DM1+P group had a lower pathologic complete response rate (44.4% vs. 55.7%; *p* = 0.016) and fewer grade ≥3 AEs (13% vs. 64%).^151^ In the long‐term follow‐up, patients receiving TDM‐1+P had a higher risk of EFS (HR: 2.61; 95% CI: 1.36–4.98) due to more locoregional preoperative progression (6.7% vs. 0%), while the risk of iDFS after surgery was similar (HR: 1.11; 95% CI: 0.52–2.40); patients in the T‐DM1+P group had fewer grade ≥3 AEs (31.8% vs. 67.7%), but a higher rate of subsequent AE‐caused treatment discontinuation (18.4% vs. 3.8%) during adjuvant setting.^152^

## 5 CHALLENGES AND PROSPECTS

The greatest advantage of ADCs is that they eliminate tumor cells, avoid healthy cells, and expand the therapeutic index.^153^ Three oncology drugs were selected as blockbuster drugs in the “2020 Cortellis Drugs to Watch,” two of which were ADCs for solid tumors, including SG for triple‐negative breast cancer patients and T‐DXd for HER2‐positive breast cancer patients.^154^ Many new ADCs are under development, such as the MMAE‐trastuzumab ADC for HER2‐positive breast cancer and rituximab‐vcMMAE ADC for CD20‐positive B‐cell lymphoma.^155^, ^156^ However, the ADC still has a huge potential for improvement.

### 5.1 Optimization of ADC structure

First, restrictively selecting extracellular antigens with highly homogeneous expression on tumors but limited expression in healthy tissues could improve the targeted selectivity of ADC and reduce drug toxicity.^12^, ^36^ However, antigens on solid tumors are highly heterogeneous and dynamic. Considering the bystander effect and non‐internalizing mechanism, the range of antigen selection is expanded because tumor cells with negative antigen expression or antigens without induction of sufficient internalization are also candidates for ADC targets, and the impact of heterogeneous antigen expression is reduced.^25^, ^157^ Potential risks include lower cellular selectivity and off‐target toxicity.^3^ Additionally, oncogenic mutant targets are potential antigens for ADCs. Mutant antigens with high and homogeneous expression have higher possibilities of ubiquitylation and internalization.^131^, ^158^ Oncogenic antigens might also avoid the downregulation of expression to elicit resistance and exert additional antitumor effects through antibody‐mediated inhibition of downstream signaling pathways.^159^, ^160^

In addition, rapid technological advancements have emerged given that the fundamental components and conjugation strategies of ADCs are significant factors that need to be improved.^161^, ^162^ Promotions of fundamental components include producing more optimized antibodies, controllable linkers, and efficient payloads. For antibody engineering, despite the advantages of IgG antibodies, partial unconjugated antibodies induce additional toxicities via ADCC and CDC, while adjustments to the ADC structure could reduce Fc gamma receptor affinity and reduce intrinsic immunological effects.^163^, ^164^, ^165^, ^166^ In addition, high molecular weight and retention in the perivascular space limit the diffusion of ADC into the tumor tissues in solid tumors.^2^, ^167^ Thus, improved antibody structures, including antibody fragments, alternative skeletons, and natural ligands, are being studied further.^168^, ^169^ Attaching payloads to small molecule fragments could improve the penetration into tumor tissues, especially tumors with poor blood supply and central nervous system tumors; however, rapid clearance remains a major problem.^170^, ^171^ In addition, the development of bispecific antibodies could enhance tumor specificity and rapid internalization, which might reduce target binding in non‐tumor tissues.^172^ As for linkers, novel metal‐mediated cleavage linkers based on simple caging moieties are under development with a well‐controlled drug release by biorthogonal bond‐cleavage reaction and a reduction in toxicity because substoichiometric amounts of metals could achieve the intended efficacy, considering the catalytic activity. Developed metal‐mediated linkers include palladium‐mediated, ruthenium‐mediated, copper‐mediated, and platinum‐mediated cleavages.^25^, ^173^ Furthermore, because of the limited number of ADCs reaching tumor cells, payloads with high efficacies are of significance, and the potential innovation of payload is not limited to cytotoxic drugs. Other agents including enzymes, protein toxins, targeted drugs, radionuclides, and immunotherapeutic drugs are also under investigation.^13^, ^174^

The conjugation strategy affects the homogeneity of the drug‐to‐antibody ratio (DAR, the average number of cytotoxic molecules attached to each antibody), the release time of cytotoxic payloads, and off‐target toxicity.^4^ Compared with traditional nonspecific conjugation, site‐specific conjugations could increase the homogeneity, stability, pharmacokinetics, and decrease toxicity.^175^, ^176^, ^177^ However, considering the inefficient chemistry and immunogenicity, new technologies are still under development for a better controlled DAR and homogeneity of ADC. For example, utilizing the dolaflexin platform, the new ADC, XMT‐1536, which targets sodium‐phosphate cotransporter protein type II (NaPi2b), is connected by a water‐soluble polymer, “Fleximer,” to improve the water‐soluble, pharmacokinetic, and immunogenicity with a DAR of 10–12. Efficacy was found with an ORR of 34% and tolerability for heavily pretreated ovarian cancer patients, which led to an FDA fast‐track designation.^178^, ^179^ Furthermore, studies have also reported computational approaches for self‐assembled synthesizing ADCs by molecular docking and dynamics simulations to overcome the instability and heterogeneity of ADCs.^180^

### 5.2 Remained challenges

Nevertheless, the development of ADCs still faces great challenges, including drug resistance and toxicity. Drug resistance remains challenging without explicit mechanisms. Current hypothetic mechanisms include decreased penetration caused by tumor microenvironment changes, downregulation of antigens, deficiencies in pathways of internalization, and resistance to payloads.^4^, ^26^ Some payloads might be transported by ATP‐binding cassette transporter proteins such as multidrug resistance protein 1 (MDR1), which induces active efflux of the payload and leads to drug resistance.^181^ Studies on resistance mechanisms could lead to promising directions for further optimization of ADCs.^147^

Toxicity is a significant factor that limits the clinical application of ADCs. Toxicity mainly depends on the positive rate and physiological function of antigens in non‐tumor tissues, the stability of linkers, the quantity and characteristics of payloads, and the bystander effect. Adverse effects of various ADCs are specific; several ADCs received black box warning, including VOD/SOS for patients receiving INO and ocular toxicity for patients receiving BM.^46^, ^182^ Adverse effects should be closely monitored, actively prevented, and timely treated with appropriate adjustments to regimens in clinical applications. Both the optimization of the ADC structure and adjustment of the administration regimen are potential solutions for reducing toxicities. The structure of ADC has been developed throughout three generations. First‐generation ADCs such as GO are mostly a combination of murine monoclonal antibodies and nondegradable linkers,^167^ which can hardly target tumor tissues accurately and fail to achieve a therapeutic effect with high toxicity.^4^, ^13^ Thus, further improvement was developed in second‐generation ADCs with improved target selectivity, reduced immunogenic humanized antibodies, more effective payloads, and stable linkers, which showed increased clinical efficacy and safety. Most of the currently approved ADCs, including BV and T‐DM1, belong to the second generation. However, disadvantages still exist, such as the presence of unbound antibodies and high DAR of ADC, which lead to off‐target toxicity, ADC aggregation, increased drug metabolic rate, and rapid clearance. Furthermore, third‐generation ADCs further optimized the previous deficiencies, including SG. Optimized site‐specific conjugation techniques, DAR of 2–4, and reduction of unbound antibodies could reduce the off‐target rate and improve the efficacy of ADCs. In addition, the fractionated dosing regimen is an approach to expand the therapeutic index, which could reduce toxicity caused by the peak concentration of ADCs in blood, extend the exposure time of ADCs in tumors, while maintaining or increasing dose intensity to ensure antitumor efficacy.^183^ For example, despite the withdrawal of GO by the FDA due to the high incidence of fatal toxicities,^184^ fractionated doses and alternative administration strategies led to FDA re‐approval with therapeutic efficacy and manageable toxicity.^40^, ^185^

### 5.3 Further exploration on clinical application

Although ADC has been widely applied in various tumor settings, most of the indications were applied to patients with treatment‐refractory cancer due to the hypoxic and immunosuppressive tumor microenvironment, hindrance of drug penetration, and the high heterogeneity of cancer.^186^ To date, all five approved ADCs for hematologic malignancies have been indicated for R/R patients, of which only BV and GO received FDA approval for first‐line treatment. In solid tumors, all four ADCs were approved for advanced previously treated patients, while only T‐DM1 was approved in the adjuvant setting. The efficacy of ADCs after resistance to traditional therapies suggests that different pathways of cytotoxic drugs and different payloads of ADCs might provide more possibilities for various sequential therapies.^29^ Other treatment settings, including first‐line therapy, are under further investigation.

Besides, despite initial exploration being mainly based on monotherapy, several ADCs showed therapeutic efficacy in combination with other drugs, including chemotherapy, targeted therapy, and immunotherapy. Cytotoxic agents with non‐overlapping mechanisms might be an option for combined therapy of ADC with chemotherapy.^187^, ^188^ The combination with targeted therapy aims to promote the overexpression or degradation of target antigens and enhance the susceptibility to ADCs, while the combination with antiangiogenic drugs might affect the efficiency of drug delivery by altering the vascular supply of the tumor.^158^, ^189^, ^190^ As for the combination of ADC and immunotherapy, ADC might increase tumor infiltrating lymphocytes (TILs) and affect the tumor microenvironment, which might improve the responses to immunotherapy.^147^, ^191^ However, the mechanisms of combined therapy, drug interactions, additive toxicities, subsequent treatments, optimal selection of patients, and further validation in clinical data are still needed.^192^, ^193^

The treatment efficacies of ADCs varied among patients, emphasizing the significance of predictive biomarkers and selection of patients. Common biomarkers are the expression and density of specific target antigens on tumor cells, which are associated with the internalization and metabolization of ADC.^194^ However, the target antigen was not sufficient to predict the efficacy of ADCs, and the detection methods and cutoff values still need to be determined. Besides, further developments of ideal biomarkers are significant, which should be able to distinguish the sensitivity of ADCs, guide treatment selections, reflect signals for early response, and monitor the therapeutic process.^2^, ^183^

## 6 CONCLUSIONS

The application of ADC has changed traditional treatment patterns for cancer patients, especially the posterior line treatment for patients with refractory tumors. Antibody drug conjugates enable the same treatment for pancreatic cancer patients and have become a great breakthrough for individualized precision treatment. Currently, with the development of ADCs, the therapeutic window is expanded and the limitation of heterogeneously expressed antigens is overcome through the bystander effect and non‐internalizing mechanism. Further exploration of indications includes patients with early stage cancer and combined therapy settings, which is of great potential. The mechanisms of drug resistance, manufacturing techniques, optimized treatment regimens, and appropriate patient selection remain as the major topics.

## CONFLICT OF INTEREST

None.

## Data Availability

Clinical data of the current study were extracted from published clinical trials, which were listed in the reference. No other date was available.
